# Supplementary material for: Are carbon emissions trading and green financial instruments synergistic? -Comprehensive quantitative research based on content analysis
Source: PLoS One. 2024 Mar 7;19(3):e0298601. doi: 10.1371/journal.pone.0298601 (PMC10919601; doi:10.1371/journal.pone.0298601)
Supplement: S1 Data — (ZIP) [file pone.0298601.s001.zip › Data/地级市Z_F工作报告.zip!/║┌┴·╜¡/╝a╬≈╩╨/└┤╫╘╣1⁄2╓┌║┼í╛┬φ┐╦╩2╛▌═°í┐_2017.docx]

各位代表：

现在，我代表市政府，向大会作报告，请予审议。

一、过去五年及2016年工作回顾

过去五年，在省委、省政府和市委的坚强领导下，我们凝聚各方力量，积极应对各种压力挑战，迎难而上，砥砺前行，经济社会实现持续健康发展。

五年来，我们以项目建设为支撑，全力实施“三大战略”，转型发展蹄疾步稳。共实施5000万元以上重点产业项目364个，累计完成投资399亿元。其中，开工建设投资10亿元以上的大项目10个，竣工投产项目214个，产业项目呈现出数量多、体量大、结构优、潜能逐步释放的发展格局。在产业项目建设牵动下，农业“两个转变”富有成效，绿色食品种植基地面积达到525万亩，比2011年增加117万亩，市级以上龙头企业达到100户；建成市级以上标准化养殖场96个，畜牧业产值占农业总产值比重比2011年提升6个百分点。工业“双轮驱动”提档增速，“两黑一绿一药”四大主导产业产值占工业比重达75%。实施企业裂变项目117个，新增规上企业71户。鸡西经济开发区晋升为省级开发区，全市各类园区入驻企业109户。我市被授予“中国石墨之都”称号，是全省首家、全国第11家矿业名城；珍宝岛药业在A股主板成功上市，开创本土企业上市先河。三产“旅贸牵动”多点发力，旅游业总收入年均增长18.3%；建成密山、虎林互市贸易区，鸡西万达广场商业综合体投入运营；现代物流业、快递业、金融保险业等加快发展；“互联网+”、“候鸟式”养老、冰雪经济等新产业、新业态、新模式竞相涌现。

五年来，我们以体制机制为保障，全力打好改革创新对上争取“组合拳”，发展动力不断增强。县级公立医院改革、医疗机构分级诊疗走在全省前列，鸡西、密山农村信用社成功改制，“营改增”试点改革、党政机关公车改革、钢铁煤炭去产能等任务基本完成。建成5个国家级、18个省级创新平台，培育创新型企业147户，实施国家、省级创新项目108个，转化各类科技成果300余项。六区独立工矿区转移支付、采沉区棚户区改造、兴凯湖流域保护、老工业基地调整改造等七大方面获得国家大力支持，资金总额达365.6亿元，对上争取的范围之广、额度之大前所未有。

五年来，我们以保障民生为根本，全力解难事办实事做好事，百姓福祉持续提升。累计民生支出427亿元，占一般公共预算支出的65%。向人民群众承诺的民生实事86件全部兑现。持续提高补助标准，城镇、企业和个体退休人员月人均待遇由2011年的1061元提高到2065元；城镇职工、居民住院报销限额分别达到13万元和9万元，城镇职工大病救助标准提高到25万元；市城乡低保实现应保尽保，标准分别比2011年提高112%和186%；市直单位住房公积金缴存比例提高到12%，达到国家上限。同时，各项社会事业取得新进步。市一中新校区、体育会展中心、市中医院新址投入使用。鸡西大学顺利升本，更名为黑龙江工业学院。建成农村综合文化中心147个、社区文化中心61个。全市459个村实现光纤全覆盖。食品药品、安全生产、社会治安持续好转。民族宗教、防灾减灾、妇女儿童、残疾人事业等各项工作均取得新成绩。

五年来，我们以生态宜居为目标，全力抓建设强功能提品质，城乡面貌大为改观。通达的交通网络进一步构建，累计建设高速公路312公里，公路总里程达到5855公里。建成穆棱河鸡西大桥等5座大型桥梁，兴凯湖机场空中航线达到6条。城市功能进一步完善，完成城市供水工程，实施城市燃气和供热管网改造工程，建成垃圾填埋厂4座、污水处理厂9座。采取PPP模式，开工建设了全省首家静脉产业园。建成穆棱河公园、城市森林公园。农村居民的生活品质进一步提升，硬化乡村街道720公里，改造农村危房2.4万户，推广分布式能源+配套卫浴一体模式1.1万户，创建市级美丽乡村示范村(达标村)125个。生态文明建设进一步加强，深度整治大气、水、土壤污染，大小兴凯湖、穆棱河水质明显改善，空气质量大幅提升。

五年来，我们以提升效能为重点，全力打造勤政为民廉洁务实政府，自身建设不断加强。围绕打造“全省最优发展环境”，实施七轮行政审批制度改革，市级行政审批由409项精简为136项，(市级非行政许可审批事项126项全部清理，梳理市级行政审批涉及的中介服务110项，保留规范84项。完成“两个清单”制定和“五证合一、一照一码”登记制度改革。在全省率先建成开通网上政务中心，成立市企业投诉中心，制定出台促进产业项目落地达产达效七项制度，从严整治不作为、慢作为、乱作为等问题，发展环境进一步优化。修改完善《鸡西市人民政府议事规则》，建立市政府系统专业知识专题学习制度，政府自身建设不断加强。五年来，共办理市人大代表议案6件，建议、批评和意见796件，市政协委员提案1249件。

2016年，是本届政府收官之年。一年来，我们全面贯彻落实习近平总书记系列重要讲话精神，以“转型发展兴市富民”为统领，担当作为，拼搏进取，较好地完成了市人大十四届七次会议确定的目标任务。

经济运行稳中向好。预计，地区生产总值增长6.5%，规模以上工业增加值增长6%，固定资产投资增长7%，社会消费品零售总额增长9%，一般公共预算收入增长5.2%，城乡居民人均可支配收入分别增长7.5%、6%。外贸进出口总额下降3.2%，好于全省平均水平。

项目建设厚积成势。全年开工建设投资5000万元以上产业项目86个，当年完成投资56.7亿元。其中，投资6.3亿元的青岛啤酒异地扩建一期、5.7亿元的福娃稻米食品产业园一期等27个项目竣工投产，为发展注入了新活力；投资7亿元的翰沃家居建材高端商场、5.9亿元的泛华货运枢纽等一批新项目开工建设，特别是投资70亿元的中韩(鸡西)石墨烯产业园项目，创造了产业项目建设体量、质量和开工速度之最，为鸡西转型发展积攒了后劲。全年招商引资新签约项目96个，当年开工91个，实际到位资金80.2亿元；国外引资项目8个，到位资金1.48亿美元。我市被列为国家国有企业退休人员社会化管理试点、增量配电业务改革试点，鸡冠区被列入国家首批产城融合示范区，恒山石墨产业园入选全国知名品牌创建示范区，虎林口岸成为全国首家进口牧草试验口岸。全年对上争取资金86亿元。

三次产业协同发展。现代农业稳步推进。新增绿色食品种植基地面积25万亩。建设省级“互联网+农业”高标准示范基地74个，农产品网上销售额6000余万元。畜牧业实现产值54亿元，增长7%。工业经济企稳回升。制定出台石墨、煤化工、生物医药、绿色食品等产业发展规划，引领产业加速成长。实施企业裂变升级项目27个，金九药业、银峰化工、德元电器等一批企业实现扩张发展。全年新增规上企业10户，拉动工业增长2.9个百分点。第三产业活力增强。珍宝岛、兴凯湖湿地荣获“黑龙江十大最美湿地”称号，虎林市晋升为国家全域旅游示范区创建试点，旅游业总收入增长19.6%。灵峰大药房登陆新三板。全市开展电子商务企业390家，网上销售额4.1亿元；快递业务量增长78.2%，收入增长71.4%。

城市功能明显增强。密兴高速公路、穆棱河众兴大桥、团结高速公路互通桥等工程竣工通车，新改扩建中心区街路17条，鸡西公路客运枢纽主体完工，增开鸡西—大连—天津空中航线，市民出行更加便捷。城市供水工程竣工通水，76.8万市民喝上优质甘甜的兴凯湖水；天然气改造工程不断扩量，4万多户居民用上清洁干净能源；改造中心区排水管网3.6公里，城市局部内涝问题得到较好解决。穆棱河、兴凯湖治理和水源地保护、大气污染防治等生态治理工程富有成效，水源地水质达标率100%，空气质量达标天数位居全省前列。

民生礼包质优量足。全年用于民生支出112.4亿元，占一般公共预算支出的65.4%。采沉区棚户区改造工程进展迅速，3.5万户居民出棚进楼，喜迁新居；贫困学生最高一次性资助标准达到5000元；首批10个贫困村、10183人全部实现脱贫，10件民生实事全部兑现。建立城乡居民大病医疗保险制度、困难残疾人生活补贴和重度残疾人护理补贴制度；在全省率先完成龙煤鸡西矿业集团第二批6397名分流人员转岗接收安置和五个矿区“两供一业”接收工作，为3.3万名困难职工发放各类救助金1000余万元；筹集资金1100万元，为全市困难群众发放节日大礼包2.1万份；三县(市)6所县级公立医院取消药品加成、降低检验检查费用，让利百姓3100万元。积极破解居民回迁、房屋证照办理、拖欠农民工工资等难题，百姓普遍关心的问题得到逐步解决。

社会事业全面进步。国家石墨产品质量监督检验中心(黑龙江)、黑龙江省石墨标准委员会、省唯一一家石墨烯院士工作站落户鸡西，建成省级石墨产业园区孵化器和中国鸡西石墨展厅。滴道、城子河、梨树、麻山通过全国义务教育均衡发展评估验收，我市被评为全国尝试教育示范实验区。建成市政务中心、公共资源交易中心、食品药品检验检测中心、农产品质量安全检验检测中心。举办了首届“青岛啤酒杯”兴凯湖公路自行车邀请赛、“珍宝岛药业杯”鸡西兴凯湖中国汽车拉力锦标赛，承办了中国乒乓球俱乐部联赛鸡西站比赛，国家水上运动赛艇集训队入驻兴凯湖训练，参训选手荣获里约奥运会铜牌。我市运动员池春雪喜获第二届冬季青奥会滑雪银牌，实现中国越野滑雪历史性突破。荣获省级卫生标兵城称号，实现“全国双拥模范城”七连冠。

各位代表！成绩来之不易，这是市委统揽全局、正确领导的结果，是市人大、市政协有效监督、鼎力支持的结果，是全市人民戮力同心、拼搏奋斗的结果。在此，我代表市政府向全市广大干部群众，向所有关心、支持鸡西发展的同志们、朋友们，表示衷心的感谢，并致以崇高的敬意！

回顾五年的实践探索，总结五年的奋斗历程，我们深刻体会到：必须坚持市委确定的发展战略不动摇，持续发力，精准发力，才能走出转型发展的新路子；必须坚持改革开放创新，促进内部活力与外部动力竞相迸发，才能培育转型发展的新动能；必须坚持共享发展理念，让发展成果更多惠及人民，才能创造兴市富民的新业绩；必须坚持依法高效行政，持续优化发展环境，才能汇聚干事创业的新合力；必须坚持实干担当奉献，困难面前不气馁，压力面前不退缩，才能在滚石上山、爬坡过坎面前展现新作为。

成绩固然令人欣慰，但我们也清醒地认识到，工作中还存在不小的差距，面临不少的问题。经济总量不大、发展速度不快、产业结构不优，是亟需解决的矛盾；四大主导产业支撑力还不强，新产业、新业态、新商业模式发展不足，创新能力偏弱，是亟需补齐的短板；立市、立县(市)区的大项目少，区级财源不足，经济增长质量有待提升，是亟需攻克的难关；创业就业困难、城镇居民收入不高、矿区人民生活环境差，以煤矿为重点的安全生产“最后一公里”还没有完全解决，是亟需破解的难题；体制机制不活，部分干部思想不够解放，“三专”能力不强，发展环境不够优化，是亟需突破的瓶颈，等等。今后，我们将坚持问题导向，探求破解路径，着力加以解决。

二、未来五年的奋斗目标和主要路径

未来五年，是鸡西全面建成小康社会的决胜期、深化改革的攻坚期、转型发展的关键期。刚刚闭幕的市第十三次党代会，描绘了鸡西未来五年的宏伟蓝图。站在新的起点上，我们将积极应对挑战，抢抓发展机遇，实干担当作为，不断开创“转型发展兴市富民”新局面。

按照市第十三次党代会确定的指导思想，今后五年政府工作的总体要求是：高举中国特色社会主义伟大旗帜，以邓小平理论、“三个代表”重要思想和科学发展观为指导，深入贯彻落实习近平总书记系列重要讲话特别是针对黑龙江重要讲话精神，按照“五位一体”总体布局和“四个全面”战略布局，牢固树立“五大发展理念”，认真落实“五大规划”和“龙江丝路带”规划，以“转型发展兴市富民”为统领，坚持突出“一条主线”，实施“三大战略”，做强“四大主导产业”，打造“一都五基地”，努力在全省率先走出资源型城市转型发展新路子，不断创造鸡西人民更加幸福美好生活。

今后五年的奋斗目标：地区生产总值年均增长8%，规模以上工业增加值年均增长10%，固定资产投资年均增长10%，社会消费品零售总额年均增长8%，一般公共预算收入年均增长6%，外贸进出口总额年均增长5%，城乡居民人均可支配收入年均都分别增长7%。

围绕上述目标，全力建设“五个鸡西”。

一是聚力转型发展，建设富裕鸡西。加快农业“两个转变”，深入推进农业供给侧结构性改革，积极构建现代农业产业、生产、经营三大体系，推动农业大市向农业强市跨越；加速工业“双轮驱动”，扩大增量，优化存量，培育“两黑一绿一药”四大百亿级产业集群，振兴实体经济；加强三产“旅贸牵动”，积极发展新业态、新模式，提高对经济增长的拉动作用，努力把鸡西打造成全省有影响力的经济强市。

二是聚力改革创新，建设活力鸡西。深入推进经济体制、行政管理体制、社会治理体制等重点领域和关键环节改革。深入实施创新驱动发展战略，推进发展动力由主要依靠资本投入向创新驱动转变。到2021年，高新技术产业增加值占GDP比重达8%。加强社会信用体系建设，基本建成法治政府，建设全省最优发展环境，努力把鸡西打造成充满生机与活力的创新创业沃土。

三是聚力合作共赢，建设开放鸡西。深度融入“龙江丝路带”，完善口岸功能，提升通关能力，建设大口岸、大物流、大通关，推动对俄合作战略升级，更广泛参与区域性、国际性合作。到2021年，外贸进出口总额实现4.5亿美元以上。坚持引资和引智、域内和域外合作并重，不断提高对外开放水平，努力把鸡西打造成“龙江丝路带”上边境旅游与贸易并举的外向型经济区。

四是聚力生态文明，建设宜居鸡西。树立绿色、协调理念，深入实施大气、水、土壤污染防治行动计划，构建绿色生态屏障，守护好蓝天碧水黑土地。加快四区一县城同城化、新型城镇化、城乡一体化步伐，努力把鸡西打造成天蓝地绿、山清水秀、功能完善、生态宜居的美丽家园。

五是聚力民生福祉，建设幸福鸡西。坚持以人民为中心的发展思想，不断提高义务教育、创业就业、社会保障、卫生健康等公共服务供给水平，打好脱贫攻坚战，加强困难群体救助，持续提高城乡居民收入，促进社会更加和谐稳定，努力把鸡西打造成人民群众安居乐业的幸福之城。

三、2017年重点工作安排

2017年，是新一届政府开局之年，是供给侧结构性改革深化之年。按照即将召开的市委经济工作会议的总体要求，牢固树立新发展理念，适应把握引领经济新常态，坚持稳中求进工作总基调，坚持以提高发展质量和效益为中心，坚持以推进供给侧结构性改革为主线，坚持以振兴实体经济为基石，深化创新驱动，培育壮大动能，激发内生动力，围绕“转型发展兴市富民”，着力建设一批重大支撑项目，扩大增量、调优存量；着力突破一批改革创新重点，转换动能、增强动力；着力办好一批民生实事好事，增进福祉、共促和谐；着力深化一批改善环境举措，优化服务、提升效能，推动经济社会平稳健康发展。

全市经济社会发展的主要预期目标为：地区生产总值增长7%，规模以上工业增加值增长8%，固定资产投资增长8%，社会消费品零售总额增长8%，一般公共预算收入增长6%，外贸进出口总额增长5%，城乡居民人均可支配收入分别增长6%、5%。节能减排指标达到省核定标准。

为实现上述目标，重点抓好以下八个方面工作。

(一)坚持数量与质量并重，在集聚项目动能上实现新突破

把产业项目建设作为“一号工程”，作为深化供给侧结构性改革有力抓手，推进产业创新升级，夯实实体经济根基。

全力推进项目建设。开展“产业项目质量效益年”活动，全年启动实施5000万元以上重点产业项目89个，当年完成投资83.8亿元。重点推进投资70亿元的中韩(鸡西)石墨烯产业园、投资50亿元的汇源生态农业综合开发、投资27.5亿元的北汽普莱德锂电负极材料和石墨深加工等7个10亿元以上大项目，尽快释放潜能；加快投资10.5亿元的贝特瑞石墨深加工、投资4.2亿元的珍宝岛鸡西二期等28个项目建设进度，力争竣工投产。坚持向质量要效益，重点建设恒辉新能源炭气油液肥联产、同泰高纯石墨等一批成长性好、科技含量高、财税见效快的“精品”项目。进一步发挥五大产业推进组统筹谋划作用，围绕国家产业政策和鸡西资源优势、产业基础，加强项目谋划，做实项目前期，增加项目储备。抓住国家实施新一轮东北振兴机遇，继续加大对上争取力度，力争普惠政策全部到位，特惠政策独享到位。

加大招商引资力度。扩大增量招商，重点推进100个线索项目、100个谋划项目招商。盘活存量招商，充分利用现有土地、厂房等闲置存量资产，进行引资嫁接、裂变升级，最大限度地释放存量动能。优化质量招商，借助“龙港经贸交流”、“台湾经贸交流”、“哈洽会”等活动，加强与国内外500强企业、行业领军企业的对接，在招商项目的数量、体量、质量上实现新突破。

提升园区建设管理水平。创新园区建设投融资机制，搭建园区投融资平台和项目开发建设平台。推行PPP模式，引进社会资本参与园区基础设施建设，重点加强鸡冠产业园区道路及配套设施，石墨产业园区污水处理厂，密山、虎林经济开发区基础建设。创新完善园区管理机制，推进园区分类管理。

(二)坚持增量与存量并重，在壮大工业实力上实现新提高

把振兴实体经济作为深化供给侧结构性改革的主要任务，深入实施“双轮驱动”战略，扩大增量，优化存量，提高质量，增加有效供给，振兴实体产业，做强实体经济。

推动石墨产业迈向中高端。以打造“中国石墨之都”为牵动，加快推进高纯石墨、锂电负极材料、石墨烯等18个深加工项目建设，提高原料就地加工转化率和增值率，推动石墨产品由初级向精深加工跃升。鼓励支持企业加强技术研发和引进，开发超细石墨粉、高导石墨块等新产品，延伸拓展石墨产业链。围绕产业链部署创新链，依托中韩石墨烯研究院、石墨烯院士工作站、石墨密封材料工程技术研究中心，加强石墨科技攻关。提升国家石墨产品质量监督检验中心能力，努力增加石墨烯、锂电池检测项目，力争国家石墨标准委员会落户鸡西，增强石墨产业发展的话语权。

壮大食品和生物医药产业。以打造绿色食品生产加工基地为牵动，围绕“粮头食尾”、“农头工尾”，大力发展主食加工、饮品加工、肉食加工业，重点突破玉米、畜产品深加工产业链，通过增品种、提品质、创品牌，提升价值链。积极引进和壮大龙头加工企业，重点推进百万吨玉米深加工、乳制品加工、纤维素燃料乙醇等项目建设。以打造生物医药制造基地为牵动，做大航母，推进珍宝岛药业三七、炎琥宁、医药集中配送等项目建设，增强龙头带动能力；做强旗舰群体，引导支持乌苏里江药业、金九药业、新医圣制药、金鑫兽药等企业，注入资本，加强合作，开发新品种，做响品牌，实现裂变升级扩张发展。推进北药产业化发展，建设鸡东、绿海林业等5个万亩北药种植基地；鼓励引导珍宝岛药业建设梨树北药交易市场。支持灵峰药业兼并扩张，鼓励小微企业联大靠强，做活做强医药商业。

积极发展提升煤炭等矿业经济。以建设现代煤化工基地为牵动，围绕“煤头电尾”、“煤头化尾”，延伸煤炭产业链。开工建设大唐热电8.5万千瓦背压机组，推动天和焦化、天诚镁业投产，持续推进中石化20亿立方米煤制气。坚持向资源开发和精深加工要发展，积极发展矿业经济，推进鸡东硅藻土、密山钾长石采选加工一体化等项目建设。实施“光伏领跑者计划”，发展壮大新能源产业，完成梨树风力发电项目建设，加快推进滴道、梨树光伏发电等项目。

推进企业裂变升级。坚持用先进技术改造传统产业，用信息化、绿色化、服务化提升现有企业，使“老字号”释放新动能，全年重点推进裂变项目20个。实施中小企业成长工程，加快推动“个转企”、“小升规”，培育壮大规上企业群体，全年新增规上企业10户。强化要素市场建设，深化银企对接，鼓励企业借助资本市场发展。支持引导企业成立产业战略联盟，影响带动企业家，强化竞争、营销、合作、规则等意识。倡导“工匠精神”，做长做久企业，打造“百年老店”。

(三)坚持品质与效益并重，在发展现代农业上实现新跨越

深入推进农业供给侧结构性改革，加快“两个转变”步伐，提高农产品供给质量，增加农业综合效益，促进农民持续增收。

推进多元化生产。把增加绿色优质农产品供给作为农业供给侧结构性改革的主攻方向，大力发展绿色品牌农业，创建农业“三减”示范区35万亩。狠抓标准化生产，继续推进“互联网+农业”高标准示范基地建设，绿色食品种植基地面积达到550万亩。把面向市场调整农业结构作为农业供给侧结构性改革的关键所在，引导近郊区大力发展棚室蔬菜、瓜果等高效设施农业，坚持“一乡一业”、“一村一品”，发展杂粮、白瓜、食用菌等特色高效作物，北药、蚕业、蜂业等特色林中经济，大白鱼、河蟹等特色水产养殖。大力发展“两牛一猪一禽”，提高标准化规模化集约化生产水平，新建千头牛场2处、万头猪场2处，创建市级标准化示范场10处。健全质量安全监管追溯体系，推进牲畜免疫无疫区建设，提高农产品质量安全监管水平。

做强产业化经营。加快土地流转，大力培育新型农业经营主体，土地适度规模经营面积达到330万亩以上。鼓励支持绿都、汇源、东粮、益海嘉里等龙头企业加快发展，增强辐射带动能力。支持引导农业合作社由生产型向生产经营型全方位转变。积极引进战略投资者，参与种养加销全过程，促进农村一二三产融合发展。

实施品牌化营销。加强品牌整合，叫响“虎林大米”、“兴凯湖大白鱼”、“神顶峰蜂蜜”等一批知名品牌，扩大鸡西绿色食品影响力。积极发展订单直销、私人订制、电子商务等现代流通方式，借助省“大米网”等电商平台，扩大网上销售，推动农产品“卖得好”，以“卖得好”带动倒逼“种得更好”。

加强社会化服务。围绕农业生产全过程，积极发展各类新型服务业态。加强虎林灌区、湖滨灌区等农田水利建设，推广农作物高产栽培技术模式，农业综合机械化程度达到96.5%。鼓励支持农民、返乡农民工、大学毕业生、退役士兵等在农业全产业链创业，全年实现农民创业3.4万人。强化精准扶贫，实现1111名贫困人口全部脱贫，提前全面完成15个村脱贫任务。

(四)坚持提质与扩容并重，在增强三产活力上实现新提升

充分发挥消费拉动经济增长的基础性作用，改善产品和服务供给，推动第三产业加快发展。

提升发展旅游业。树立“全域发展、全景鸡西”理念，实施“旅游+”工程，推动旅游跨界融合发展，全力打造生态度假边境观光旅游基地。丰富产品供给，推进兴凯湖景区、虎头景区规范管理，提升景区档次。支持虎头珍宝岛景区晋升5A级景区，创建虎林全国红色旅游国际合作区、密山边境旅游试验区。打造经典线路，加强域内外旅行社合作，统筹域内旅游资源，集中打造生态观光、避暑养生、红色文化、跨境康体、冰雪之旅等精品线路。持续加强营销，建立“龙江东部旅游产业联盟”，加强与龙广传媒、同程、途牛的合作，不断提升鸡西旅游影响力和知名度，叫响“美丽鸡西——不一样的江湖”品牌。完善精细管理，加强景区交通、宾馆等基础设施建设，提高旅游接待能力和服务水平，规范旅游市场。全年接待国内外游客人数、旅游业总收入分别增长15%以上。

繁荣活跃商贸业。积极融入省“龙江丝路带”建设，全力打造对俄国际通道和进出口加工基地。面向国内国外两个市场，加强特色专业口岸建设，扩大石化产品、粮食、水生动物、肉制品、中草药、木材等进口，提高蔬菜、啤酒、机电等地工产品出口比重。推进虎林中草药口岸获批。支持双叶、大济、远华、金果等外贸企业发展，做强外贸主体。进一步活跃口岸互市贸易区，争创密山、虎林沿边边境经济合作区，务实推动对俄教育、科技、文化等多领域全方位交流合作。加快发展物流业，推进泛华货运枢纽、翰沃商贸城等项目建设，打造区域性物流中心。改造提升传统商贸业，鼓励支持限上企业、主流超市、专业批发市场，开展“线上线下”互动的网络零售业务。

积极发展新业态。制定出台《鸡西市促进电子商务发展若干扶持政策》。完善提升鸡冠区、虎林电子商务园区和孵化基地功能，支持石墨、煤海、绿色食品、跨境旅游等交易平台建设，全年电子商务交易额突破6亿元。加快鸡冠区养老服务中心建设，发展“医养结合”、“互联网+养老”新模式，打造环兴凯湖候鸟式旅居养老服务基地。办好兴凯湖国际马拉松、冰雪汽车挑战赛、乌苏里江南岛湖国际冰钓大赛等体育赛事，促进体育产业发展。加快发展冰雪旅游、冰雪体育、冰雪文化等冰雪经济，把“冷资源”变成“热产业”。积极培育文化、培训、会展、信息服务等幸福产业和新兴服务业。

(五)坚持改革与创新并重，在激发内生动力上实现新增强

推进重要领域和关键环节改革，实施创新驱动战略，持续优化发展环境，激发市场活力，增强发展动力。

全面深化改革。深化行政管理体制改革，动态调整权力清单、责任清单、中介服务清单，启动制定负面清单；坚持市场化配置公共资源，试点推行政府购买服务改革；完成国有林场改革；启动供热费补贴方式改革；有序推进事业单位分类改革。深化经济体制机制改革，加大供给侧结构性改革力度，落实“三去一降一补”重点任务，研究制定降低企业制度性成本的意见措施；积极推进增量配电业务改革、厂办大集体改革；完成土地承包经营权确权登记工作。深化社会管理体制改革，推进安全生产领域改革，启动出租车行业改革，全面推开城市公立医院综合改革。

深入推进创新驱动。实施“千户科技型企业三年行动计划”，新创办科技型企业80户，高新技术产业产值达60亿元以上。新建院士专家工作站、省级重点技术研究中心3户，建设科技企业孵化器、众创空间等创新平台41处。实施石墨烯传感器开发、宝石级金刚石产业化开发、洁净动力配煤技术等重大科技项目。依托黑龙江石墨产业技能人才培训基地和黑龙江工业学院，培养一批“专业工匠”、“技术达人”和创新人才；制定完善人才引进、股权激励、成果转化等系列政策，推动科技人才兼职兼薪、离岗创业；加强外来人才专家公寓建设，用事业和待遇吸引留住人才。

持续优化发展环境。以打造全省最优发展环境为目标，推进“放管服”工作，提高政府的行政效率和服务水平。发挥市公共资源交易中心、网上政务中心、智慧城市“一号通”作用，创造更加便民便企的服务环境。加强与龙煤、农垦、森工等国有企业合作，促进民营经济发展，推进各类市场主体公平竞相发展。加强政务诚信、商务诚信、社会诚信建设，强化金融监管，防范金融风险，优化金融环境。聚焦企业和群众反映的突出问题，整治损害经济发展环境行为，构建“亲”“清”新型政商关系，打造法治、公平、透明的营商环境。

(六)坚持建设与管理并重，在提高城乡品质上实现新进步

坚持完善功能，提升品质，统筹推进城乡建设，全力打造美丽生态宜居家园。

推进同城化建设。坚持规划引领，做好海绵城市、地下管廊、雨水防涝、城市停车场、通讯基础设施等专项规划。围绕“一河居中、两岸同兴，东跨南进、西拓北连”的发展思路，谋划实施滴道、城子河外环路建设，推进鸡冠区、恒山区、滴道区、城子河区、鸡东县城道路交通互联、基础设施共建、产业协同发展，构建“四区一县城”主城区发展格局。启动鸡冠产城融合示范区建设，优化中心区空间布局，增强辐射带动能力。

统筹城乡一体化建设。完成牡佳客运专线鸡西段征收搬迁，改造升级鸡图路，新改扩建中心区街路20条。完成文化西路等10处园林工程、东山街等4条街路路灯改造工程和永昌东路等7处排水工程。开工建设中水处理厂，改造老旧供热管网10公里，建成中燃燃气永久场站，完成静脉产业园建设。加快虎林机场前期工作。推动密山、虎林、鸡东中心城镇改造和延展，提升聚集人口能力；加强梨树、麻山卫星城镇建设，打造虎头、白鱼湾等一批旅游名镇、特色小镇。实施分布式能源建设，创建一批省级美丽乡村示范村，让城乡居民共同享受高品质生活。

加强生态环境建设。完成兴凯湖和穆棱河流域36个生态保护项目建设，持续改善流域水质，让鸡西的水更清。加大燃煤小锅炉和黄标车淘汰力度，严格重点企业污染治理设施运行监管，推进秸秆限烧禁烧和综合利用，让鸡西的空气更清新。实施乌苏里江、兴凯湖堤岸治理工程，推进土壤污染防治，加强采沉区生态修复，加大退耕还林、还湿力度，让鸡西的生态更优良。

提高城市管理水平。着力加强市容市貌管理，重点整治乱贴乱画乱放，规范停车秩序、户外广告牌匾，提升重点街路全天候保洁水平，持续开展背街巷道、城市出入口的环境卫生整治。着力加强物业管理，改造南山、新华等15个老旧小区，达到物业企业入驻标准，发挥业主委员会作用，引入退出机制，提升小区物业管理水平。着力加强城市智能化管理，稳步推进城市综合执法改革，建立联席会议制度，健全信息共享、部门协作、反应迅速的联动机制。推进智慧城市管理平台建设，提高城市执法、市政环卫、园林物业、城市交通等领域智能化管理水平。着力加强城市文明建设，以创建全国文明(提名)城市为目标，大力倡导文明之风，积极开展争做文明机关、文明学校、文明企业、文明市民活动，让文明成为习惯，树立文明鸡西形象。

(七)坚持扩面与提质并重，在增进民生福祉上实现新作为

顺应人民群众对幸福美好生活的新期待，加大力度补齐民生短板，让百姓共享改革发展成果。

倾力办好10件民生实事。(1)全面完成采沉区棚户区三年改造任务，剩余3.2万户采沉区居民全部出棚进楼。(2)实施8个破产矿区、5个独立工矿区“三供一业”改造工程，改善矿区20万群众生活环境，提高生活质量。(3)实施农村分布式能源+配套卫浴一体模式5000户，提升农村居民生活品质。(4)创造条件，集中力量解决已入住楼房居民不能办理产权证问题。(5)开工建设鸡冠腾飞小区标准化学校等9个项目，满足学生就近入学需求。加强扶贫助学，全年资助贫困学生3.1万人次。(6)开工建设鸡西福利性精神病医院和鸡西老年养护院，提高城乡特困群体救助水平。(7)实施重点人群“家庭医生”计划，实现普惠群众23万人。(8)开工建设铁电街跨铁立交桥，年内竣工通车，畅通城市交通。(9)更新市区70%以上公交车，总量达500台以上。按需求向建成小区延伸公交线路，方便群众出行。(10)建设开通城市“一号通”公共服务系统，让百姓办事更便捷、更便利。

提升社会保障能力。全力做好创业就业工作，落实创业各项优惠政策，加强众创空间、孵化器建设，推动科技人员、大学生、农民、龙煤转移分流职工四支队伍创新创业。坚持以创业带动就业，加强创业孵化基地建设，提升创业就业能力。全年城镇新增就业3.2万人，城镇登记失业率控制在4.6%以下。提升社会保障水平，适度提高机关事业单位和企业退休人员基本养老金水平，落实整合城乡居民基本医疗保险制度，实现“覆盖范围、筹资政策、保障待遇、医保目录、定点管理、基金管理”六统一。加强社会救助，提高城乡低保和特困人员供养水平，稳步扩大企业困难职工、受灾群众的救助范围。

推动社会事业全面进步。推进义务教育均衡发展，新建、续建校舍3.9万平方米，实现国家“义务教育发展基本均衡县(市)区”全覆盖。实施“文化惠民工程”，扩大地面无线数字电视覆盖范围，加强农村文化活动室和城市社区文化中心建设。积极发展卫生与健康事业，编制《“健康鸡西2030”规划纲要》，推进公立医院与基层医疗卫生机构分级诊疗、双向转诊，开展医疗卫生精准扶贫计划，贫困人口全部纳入重特大疾病救助范围。建成标准化社区15个，进一步完善功能，提高服务水平。

全力维护安全稳定。突出抓好以煤矿为重点的安全生产工作，切实解决“最后一公里”问题，促进安全生产形势持续稳定。统筹推进非煤矿山、交通运输、消防森防、危险化学品、烟花爆竹、特种设备、食品药品等重点领域以及学校、商场等人员密集场所的安全。落实信访属地管理责任，加大领导干部接访、下访力度，畅通信访渠道，妥善解决群众合理诉求，坚持依法治访，坚决打击闹访、缠访等违法行为。加强边境安全管控，强化社会治安综合治理，构建立体化防控体系，保持对各类违法犯罪活动的高压态势，提高人民群众的安全感。

(八)坚持法治与效能并重，在政府自身建设上实现新加强

完成2017年工作任务，实现未来五年的奋斗目标，政府肩负着重要的责任，唯有忠于职守、履职尽责、依法高效、勤政清廉，才能不辱使命，不负重托。

坚持忠诚为民履职。切实增强“四个意识”，努力建设对党忠诚、为人民负责的政府公务员队伍。大力倡导解放思想，坚决破除影响和制约鸡西发展的思想观念羁绊，以先行抢占先机，以先试探索新路。深化“三专”公务员队伍建设，进一步完善政府系统学习制度，提升广大公务员适应把握引领经济发展新常态的能力，始终保持与时俱进、锐意改革、开拓创新的精神风貌，挺起转型发展的精气神。

坚持实干担当尽责。大力倡导敢闯敢试、敢于碰硬、敢于承重的风气，撸起袖子加油干，铸就推动发展的“铁肩膀”。把抓落实作为衡量担当成效的重要标准，继续大力倡导“四办”作风，健全完善抓落实的工作机制，对看准的事情、定下的目标、部署过的工作，雷厉风行、一抓到底，推动各项工作干在实处、走在前列。

坚持依法高效行政。定期向党委、人大汇报工作，向政协通报情况，自觉接受人大法律监督和政协民主监督，努力提高办理人大代表议案建议和政协提案的质量。强化法治思维和依法履职意识，建立健全公众参与、专家论证、政府集体决定的决策机制，让权力在阳光下运行。坚持以信息化技术、互联网思维创新政府服务管理，让数据“多走路”、群众“少跑腿”，促进服务和效率“双提升”、职能和作风“双转变”。

坚持廉洁干净干事。严格落实全面从严治党的各项纪律要求，切实履行主体责任，落实“一岗双责”，遵守“六大纪律”，营造风清气正的干事环境。严格监督检查，围绕国有土地资源出让、工程招投标、财政资金使用等重点领域，加大行政监察和财政、审计监督力度。厉行勤俭、反对浪费，严格控制一般性支出，确保“三公”经费只减不增，打造廉洁政府。

各位代表，不忘初心，勇往直前，梦想终会实现。站在新的历史起点上，让我们紧密团结在以习近平同志为核心的党中央周围，在市委的坚强领导下，团结一心，真抓实干，奋发有为，为开创转型发展兴市富民新局面，创造鸡西人民更加幸福美好生活而努力奋斗！
